# Synthesis and crystal structure of the cluster (Et_4_N)[(Tp*)MoFe_3_S_3_(μ_3_-NSiMe_3_)(N_3_)_3_]

**DOI:** 10.1107/S2056989024004833

**Published:** 2024-05-31

**Authors:** Yue Li, Jia Wei, Jie Han, Xu-Dong Chen

**Affiliations:** a Nanjing Normal University, 1 Wenyuan Road, Qixia district, Nanjing, Jiangsu 210023, People’s Republic of China; b School of Science and Technology, Hong Kong Metropolitan University, Hong Kong; Harvard University, USA

**Keywords:** crystal structure, Mo–Fe–S cluster, FeMo cofactor, synthesis

## Abstract

The title compound, tetra­ethyl­ammonium tri­azido­tri-μ_3_-sulfido-[μ_3_-(tri­methyl­sil­yl)aza­nediido][tris­(3,5-di­methyl­pyrazol-1-yl)hydro­borato]triiron(+2.33)molybdenum(IV), (C_8_H_20_N)[Fe_3_MoS_3_(C_15_H_22_BN_6_)(C_3_H_9_NSi)(N_3_)_3_] or (Et_4_N)[(Tp*)MoFe_3_S_3_(μ_3_-NSiMe_3_)(N_3_)_3_] [Tp* = tris­(3,5-di­methyl­pyrazol-1-yl)hydro­bor­ate(1−)], crystallizes as needle-like black crystals in space group *P*  . In this cluster, the Mo site is in a distorted octa­hedral coordination model, coordinating three N atoms on the Tp* ligand and three μ_3_-bridging S atoms in the core. The Fe sites are in a distorted tetra­hedral coordination model, coordinating two μ_3_-bridging S atoms, one μ_3_-bridging N atom from Me_3_SiN^2−^, and another N atom on the terminal azide ligand. This type of heterometallic and heteroleptic single cubane cluster represents a typical example within the Mo–Fe–S cluster family, which may be a good reference for understanding the structure and function of the nitro­genase FeMo cofactor. The residual electron density of disordered solvent mol­ecules in the void space could not be reasonably modeled, thus the SQUEEZE [Spek (2015). *Acta Cryst*. C**71**, 9–18] function was applied. The solvent contribution is not included in the reported mol­ecular weight and density.

## Abstract 2

This type of heterometallic and heteroleptic single cubane cluster represents a typical example within the Mo–Fe–S cluster family, which may be a good reference for understanding the structure and function of the nitro­genase FeMo cofactor.

## 1. Chemical context

Nitro­gen is abundant in the atmosphere in the form of di­nitro­gen gas, but this type of nitro­gen cannot be metabolized by organisms directly (Jia & Quadrelli, 2014 ▸; MacKay & Fryzuk, 2004 ▸). It must be fixed by nitro­genase in some selected microorganisms (Dos Santos *et al.*, 2012 ▸). Nitro­genase can transform N_2_ to NH_3_, and then the biochemical N cycle sets off (Cheng, 2008 ▸; Canfield *et al.*, 2010 ▸). The exploration of synthetic structural analogs of nitro­genase is therefore a crucial area in modern science research.

The FeMo cofactor is believed to be one of the most important parts in nitro­genase responsible for nitro­gen fixation. The FeMo cofactor contains a 2*p* atom in the center, which has been proven to be a carbide, resulting in the structure as [MoFe_7_S_9_C] (Spatzal *et al.*, 2011 ▸; Lancaster *et al.*, 2011 ▸). To mimic the structure of the FeMo cofactor, a large number of iron–sulfur clusters have been synthesized (Lee & Holm, 2004 ▸; Holm, 1977 ▸; Herskovitz *et al.*, 1972 ▸; Liu *et al.*, 1990 ▸; Nordlander *et al.*, 1993 ▸). However, synthesizing heteroleptic analogs with a 2*p* atom in the core of the cluster is a tough challenge for researchers in this area (Sickerman *et al.*, 2017 ▸). With the unremitting efforts of scientists, some synthetic homometallic or heterometallic iron–sulfur clusters with a 2*p* atom in the core have been synthesized. Lee’s group have used the dinuclear precursors for the selective synthesis of the homometallic cubane clusters [Fe_4_(N^*t*^Bu)_*n*_(S)_4–*n*_Cl_4_]^*z*^ with (*n*, *z* = 3, 1−, 2, 2− or 1, 2−; Chen *et al.*, 2010 ▸). Our group have developed core ligand metathesis and core ligand redox metathesis strategies and successfully synthesized versatile heterometallic iron–sulfur clusters containing a core 2*p* atom, including the [*M*Fe_3_S_2_(μ_2_-*Q*)]^1+^ and [*M*Fe_3_S_3_(μ_3_-*Q*)]^2+^ (*M* = W and Mo, *Q* = N*R*, O*R*) cubane clusters (Xu *et al.*, 2018 ▸; He *et al.*, 2022 ▸), and the [(Tp*)_2_W_2_Fe_6_(μ_4_-N)_2_S_6_ *L*_4_]^2−^ [Tp* = tris­(3,5-di­methyl­pyrazol-1-yl)-hydro­borate(1−), *L* = Cl^−^ or Br^−^] double cubane clusters (Xu *et al.*, 2019 ▸). Previously in our laboratory, the molybdenum–iron–sulfur cluster [(Tp*)MoFe_3_S_3_(μ_3_-NSiMe_3_)Cl_3_]^−^, which resembles one of the cubic subunits of the FeMo cofactor, was synthesized through a LEGO-like strategy. Based on this cluster, which has a μ_3_-bridging N atom in the core, we explored the effects of terminal ligands on the Fe sites of heterometallic heteroleptic iron–sulfur clusters. In this work, terminal ligand substitution using NaN_3_ was applied to produce the cluster [(Tp*)MoFe_3_S_3_(μ_3_-NSiMe_3_)(N_3_)_3_]^−^. The synthesis and structural analysis of this compound may provide useful information for a better understanding of the structure and reactivity of the FeMo cofactor, as well as how the terminal ligand affects the physical property of the cluster (Xu *et al.*, 2018 ▸; He *et al.*, 2022 ▸). 
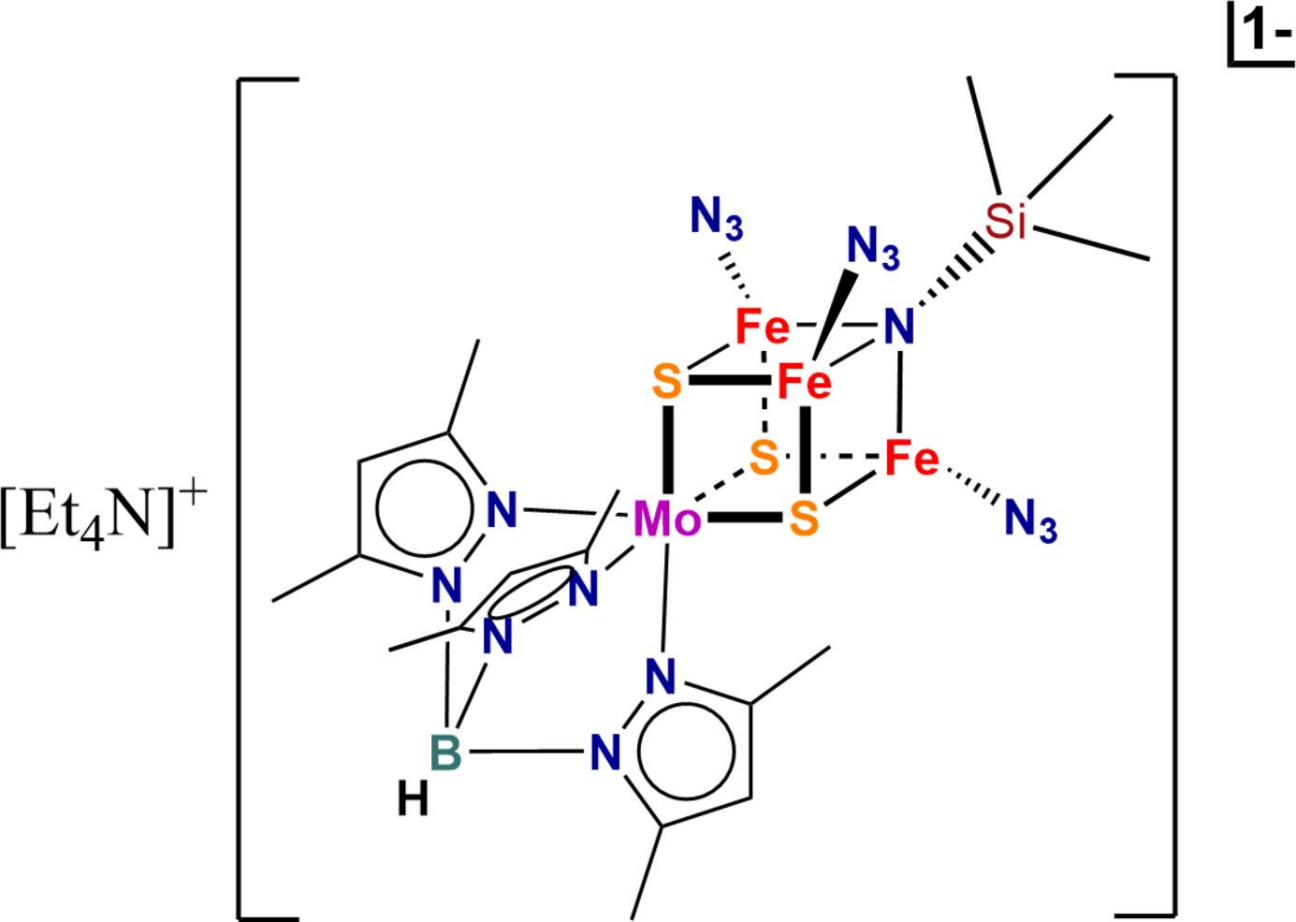


## 2. Structural commentary

**Figure 1:**
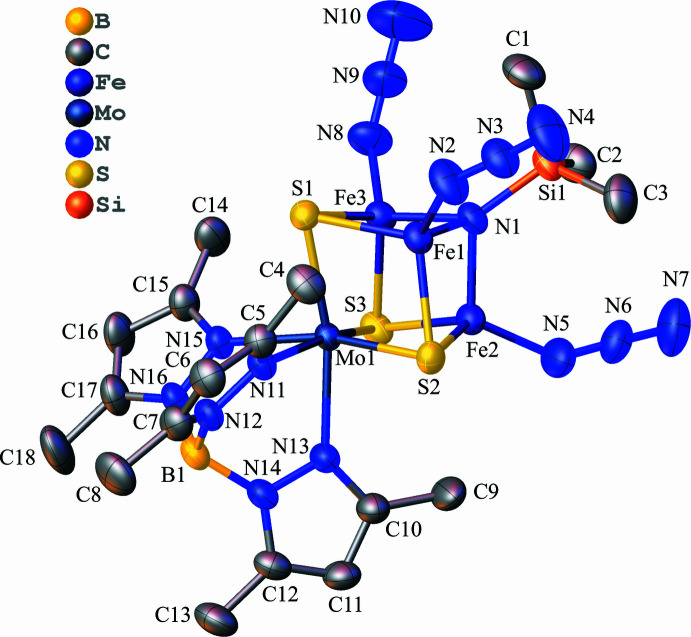
Structure of the anionic cluster in the title compound with the atom-numbering scheme. Displacement ellipsoids are drawn at the 50% probability level. Hydrogen atoms are omitted for clarity.

This title cluster crystallized as the Et_4_N^+^ salt in the triclinic crystal system, space group *P* 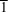 . The different metal atoms exhibit distinct coordination models in this cluster. The Mo site coordinates three N atoms of the Tp* ligand and three μ_3_-bridging S atoms in the core of the cluster, showing a distorted octa­hedral coordination sphere. Each Fe site coordinates two μ_3_-bridging S atoms, one μ_3_-bridging N atom from Me_3_SiN^2−^, and one N atom on the terminal ligand, resulting in a distorted tetra­hedral geometry. The cluster exhibits quasi-threefold symmetry in its crystal form, as a result of the steric constraint generated by the crystal packing. In the core of the cluster, the Mo—S bond lengths range from 2.3638 (13) to 2.3758 (14) Å, with an average value of 2.369 (2) Å. The Mo⋯Fe distances are between 2.7743 (12) Å and 2.8012 (13) Å, averaging 2.789 (1) Å. The Fe⋯Fe distances fall in the range 2.6123 (12) Å and 2.6368 (11) Å, with a mean value of 2.626 (1) Å. The Fe—S bond lengths range from 2.2678 (14) to 2.2923 (13) Å, with an average value of 2.282 (1) Å. The Fe—N(imide) bond lengths are in the range of 1.917 (2) Å to 1.9386 (19) Å, with an average value of 1.931 (2) Å. The Fe—N(azide) bond lengths are between 1.922 (2) and 1.937 (2) Å, with an average value of 1.930 (2) Å. The N—Si bond length is 1.753 (2) Å. The Fe—N—Fe angles range from 84.78 (7) to 86.29 (7)° with an average of 85.7 (1) °. The structure of the cluster [(Tp*)MoFe_3_S_3_(μ_3_-NSiMe_3_)(N_3_)_3_]^−^ is shown in Fig. 1 ▸ and some selected geometric parameters are listed in Table 1 ▸.

## 3. Supra­molecular features

**Figure 2:**
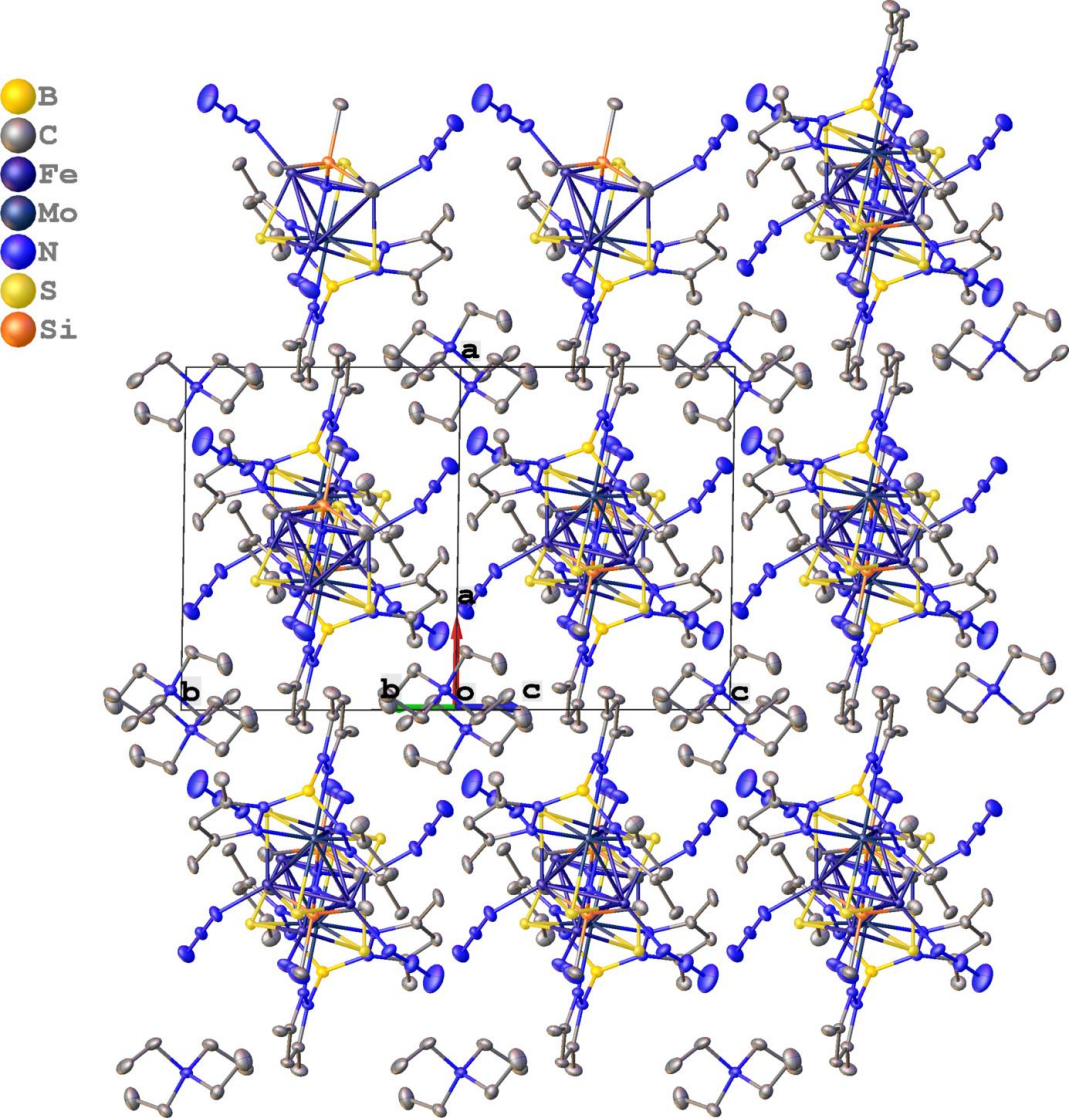
Crystal packing of the title compound. Hydrogen atoms are omitted for clarity.

In the crystal, there are two sets of cluster counter-ions in each unit cell. The anionic clusters and the Et_4_N^+^ cations are arranged in alternating layers, where electrostatic inter­actions might be the dominant supra­molecular inter­actions. No significant hydrogen-bonding or π–π stacking inter­actions were identified in the crystal structure. The packing of the title compound is shown in Fig. 2 ▸.

## 4. Database survey

Heteroleptic cubane-type *M*–Fe–S–N clusters (*M* = Mo or W) are very rare. In the literature, there are currently only two types of *M*–Fe–S–N clusters (Xu *et al.*, 2018 ▸; He *et al.*, 2022 ▸; Zhang *et al.*, 2023 ▸). Thus far, cubane-type Mo–Fe–S–N clusters with azide terminal ligands have not been synthesized successfully.

A search of the Cambridge Structural Database with WebCSD (updated to November 2023; Groom *et al.*, 2016 ▸) revealed two types of heteroleptic cubane-type *M*–Fe–S–N clusters (*M* = Mo, W), *viz*. [(Tp*)WFe_3_S_3_(μ_3_-NSiMe_3_)*L*_3_]^−^ [NIFBIQ (*L*= Cl^−^); Xu *et al.*, 2018 ▸; XIGKEH, XIGKAD, XIGKOR, XIGKIL, XIGKUX (*L* = SMe^−^, SEt^−^, SPh^−^, SPhMe^−^, N_3_^−^); Zhang *et al.*, 2023 ▸] and [(Tp*)MoFe_3_S_3_(μ_3_-NSiMe_3_)Cl_3_]^−^ (RAWLAG; He *et al.*, 2022 ▸).

## 5. Synthesis and crystallization

**Figure 3:**
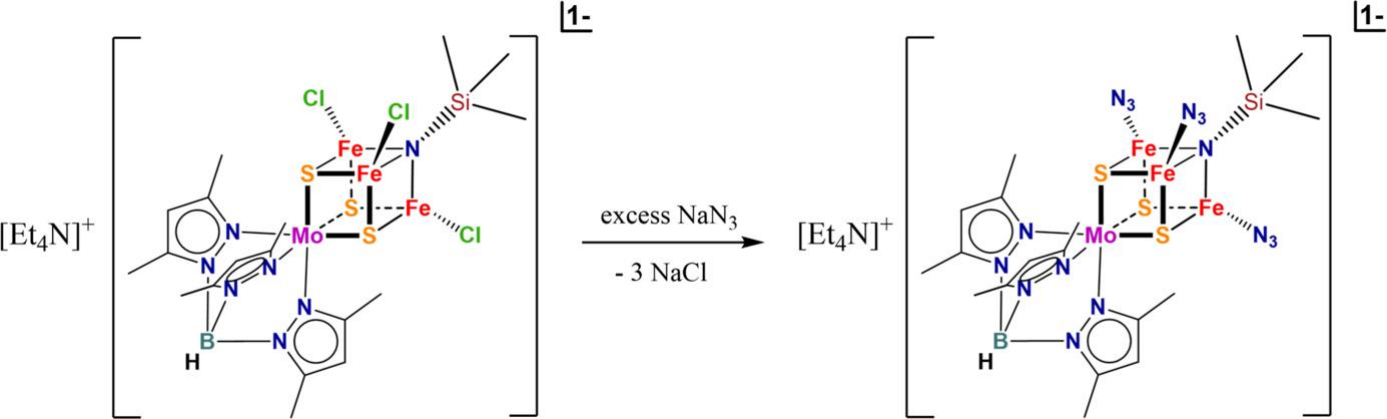
Synthesis of (Et_4_N)[(Tp*)MoFe_3_(μ_3_-S)_3_(μ_3_-NSiMe_3_)(N_3_)_3_].

All reactions and manipulations were performed in a glovebox under an atmosphere of dry N_2_. DMF was refluxed over CaH_2_ until dry and was distilled under an N_2_ atmosphere. Diethyl ether was refluxed over sodium metal and benzo­phenone until dry and was distilled under an N_2_ atmosphere. All solvents were stored in a glovebox over activated mol­ecular sieves (3 Å). NaN_3_ was stored in a glovebox under an atmosphere of dry N_2_. As shown in Fig. 3 ▸, NaN_3_ (7.8 mg, 0.12 mmol) was added into a DMF solution (3.0 mL) of (Et_4_N)[(Tp*)MoFe_3_(μ_3_-S)_3_(μ_3_-NSiMe_3_)Cl_3_] (29.4 mg, 0.03 mmol). After overnight stirring, the color of the reaction mixture changed to brownish yellow. Filtration was done through celite and the filtrate was diffused by diethyl ether at room temperature to give needle-like black crystals (10.9 mg, yield: 36%). ^1^H NMR (DMSO-*d*_6_, 400 MHz, δ, ppm): 5.83 (*s*, 3H, CH), −0.01 (*s*, 9H, CH_3_), −8.15 (*vbr*, 9H, CH_3_). Other proton signals could not be located due to paramagnetic broadening. Elemental analysis: calculated for C_26_H_51_BFe_3_MoN_17_S_3_Si: C, 31.22; H, 5.14; N, 23.80. Found: C, 31.73; H, 5.35; N, 23.27. IR (cm^−1^): ν (N=N), 2059 (*vs*). UV (nm) λ: 245, 345, 555.

## 6. Refinement

Crystal data, data collection, and structure refinement details are summarized in Table 2 ▸. Hydrogen atoms were added at idealized positions and refined using a riding model. The residual electron density of disordered solvent mol­ecules in the void space could not be reasonably modeled, thus the SQUEEZE (Spek, 2015 ▸) function was applied in *PLATON* (Spek, 2020 ▸). A total of 40 electrons in a volume of 146 Å^3^ were counted by SQUEEZE and removed per unit cell. This accounts for about one solvent mol­ecule (probably diethyl ether) per unit cell.

## Supplementary Material

Crystal structure: contains datablock(s) I. DOI: 10.1107/S2056989024004833/oi2008sup1.cif

Structure factors: contains datablock(s) I. DOI: 10.1107/S2056989024004833/oi2008Isup2.hkl

IR and UV data. DOI: 10.1107/S2056989024004833/oi2008sup3.docx

CCDC reference: 2353398

Additional supporting information:  crystallographic information; 3D view; checkCIF report

## Figures and Tables

**Table 1:** Selected geometric parameters (Å, °)

Mo1—Fe1	2.7743 (12)	Fe1—N2	1.937 (2)
Mo1—Fe2	2.8012 (13)	Fe2—Fe3	2.6286 (11)
Mo1—Fe3	2.7920 (11)	Fe2—S2	2.2906 (14)
Mo1—S1	2.3660 (15)	Fe2—S3	2.2923 (13)
Mo1—S2	2.3638 (13)	Fe2—N1	1.917 (2)
Mo1—S3	2.3758 (14)	Fe2—N5	1.932 (2)
Fe1—Fe2	2.6368 (12)	Fe3—S1	2.2824 (12)
Fe1—Fe3	2.6123 (12)	Fe3—S3	2.2784 (14)
Fe1—S1	2.2794 (14)	Fe3—N1	1.936 (2)
Fe1—S2	2.2678 (14)	Fe3—N8	1.922 (2)
Fe1—N1	1.9386 (19)	Si1—N1	1.7530 (19)
			
Fe1—N1—Fe2	86.29 (7)	Fe2—N1—Fe3	86.02 (7)
Fe1—N1—Fe3	84.78 (7)		

**Table 2:** Experimental details

Crystal data	
Chemical formula	(C_8_H_20_N)[Fe_3_MoS_3_(C_15_H_22_BN_6_)(C_3_H_9_NSi)(N_3_)_3_]
*M*_r_	1000.40
Crystal system, space group	Triclinic, *P* 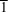
Temperature (K)	296
*a*, *b*, *c* (Å)	10.689 (6), 11.321 (6), 19.030 (11)
α, β, γ (°)	75.306 (7), 84.362 (7), 86.829 (7)
*V* (Å^3^)	2216 (2)
*Z*	2
Radiation type	Mo *K*α
μ (mm^−1^)	1.45
Crystal size (mm)	0.02 × 0.01 × 0.01

Data collection	
Diffractometer	Bruker APEXII CCD
Absorption correction	Multi-scan (*SADABS*; Krause *et al.*, 2015 ▸)
*T*_min_, *T*_max_	0.615, 0.746
No. of measured, independent and observed [*I* > 2σ(*I*)] reflections	31083, 10181, 8508
*R*_int_	0.022
(sin θ/λ)_max_ (Å^−1^)	0.654

Refinement	
*R*[*F* ^2^ > 2σ(*F* ^2^)], *wR*(*F* ^2^), *S*	0.027, 0.071, 1.02
No. of reflections	10181
No. of parameters	482
No. of restraints	36
H-atom treatment	H-atom parameters constrained
Δρ_max_, Δρ_min_ (e Å^−3^)	0.34, −0.28

Computer programs: *APEX2* and *SAINT* (Bruker, 2016 ▸), *SHELXT2018/2* (Sheldrick, 2015*a* ▸), *SHELXL2018/3* (Sheldrick, 2015*b* ▸) and *OLEX2* (Dolomanov *et al.*, 2009 ▸).

## supplementary crystallographic information

### Crystal data

**Table:**

(C_8_H_20_N)[Fe_3_MoS_3_(C_15_H_22_BN_6_)(C_3_H_9_NSi)(N_3_)_3_]	*Z* = 2
*M**_r_* = 1000.40	*F*(000) = 1026
Triclinic, *P*1	*D*_x_ = 1.500 Mg m^−^^3^
*a* = 10.689 (6) Å	Mo *K*α radiation, λ = 0.71073 Å
*b* = 11.321 (6) Å	Cell parameters from 9959 reflections
*c* = 19.030 (11) Å	θ = 2.3–27.5°
α = 75.306 (7)°	µ = 1.45 mm^−^^1^
β = 84.362 (7)°	*T* = 296 K
γ = 86.829 (7)°	Needle, dark black
*V* = 2216 (2) Å^3^	0.02 × 0.01 × 0.01 mm

### Data collection

**Table:**

Bruker APEXII CCD diffractometer	10181 independent reflections
Radiation source: sealed tube	8508 reflections with *I* > 2σ(*I*)
Graphite monochromator	*R*_int_ = 0.022
Detector resolution: 8 pixels mm^-1^	θ_max_ = 27.7°, θ_min_ = 1.9°
φ and ω scans	*h* = −13→13
Absorption correction: multi-scan (SADABS; Krause *et al.*, 2015)	*k* = −14→14
*T*_min_ = 0.615, *T*_max_ = 0.746	*l* = −24→24
31083 measured reflections	

### Refinement

**Table:**

Refinement on *F*^2^	36 restraints
Least-squares matrix: full	Hydrogen site location: inferred from neighbouring sites
*R*[*F*^2^ > 2σ(*F*^2^)] = 0.027	H-atom parameters constrained
*wR*(*F*^2^) = 0.071	*w* = 1/[σ^2^(*F*_o_^2^) + (0.0361*P*)^2^ + 0.6048*P*] where *P* = (*F*_o_^2^ + 2*F*_c_^2^)/3
*S* = 1.02	(Δ/σ)_max_ = 0.002
10181 reflections	Δρ_max_ = 0.34 e Å^−^^3^
482 parameters	Δρ_min_ = −0.28 e Å^−^^3^

### Special details

**Table:**

Geometry. All esds (except the esd in the dihedral angle between two l.s. planes) are estimated using the full covariance matrix. The cell esds are taken into account individually in the estimation of esds in distances, angles and torsion angles; correlations between esds in cell parameters are only used when they are defined by crystal symmetry. An approximate (isotropic) treatment of cell esds is used for estimating esds involving l.s. planes.
Refinement. Single-crystal X-ray diffraction data for the title compound was collected at 296 K on a Bruker APEX II CCD diffractometer operating at 50 kV and 30 mA using Mo-Kα radiation (λ = 0.71073 Å). Crystal was mounted on a loop using Parabar 10312 oil for data collection. Data was collected with a series of φ and/or ω scans. Data was integrated using SAINT and scaled with either a numerical or multiscan absorption correction using SADABS. Structure was solved using SHELXT and refined by full-matrix least-squares on *F*^2^ using the SHELXL and OLEX2 (Dolomanov *et al.*, 2009) programs. All non-hydrogen atoms were refined anisotropically.

### Fractional atomic coordinates and isotropic or equivalent isotropic displacement parameters (Å^2^)

**Table:**

	*x*	*y*	*z*	*U*_iso_*/*U*_eq_	
Mo1	0.37687 (2)	0.19344 (2)	0.70187 (2)	0.02871 (5)	
Fe1	0.35280 (3)	0.34195 (3)	0.79844 (2)	0.03469 (7)	
Fe2	0.51034 (3)	0.15159 (3)	0.82587 (2)	0.03669 (8)	
Fe3	0.56554 (3)	0.34312 (3)	0.71799 (2)	0.03547 (8)	
S1	0.37571 (5)	0.40934 (5)	0.67454 (3)	0.03587 (11)	
S2	0.29666 (5)	0.14413 (5)	0.82569 (3)	0.03783 (12)	
S3	0.59472 (5)	0.14523 (5)	0.71135 (3)	0.03843 (12)	
Si1	0.59645 (6)	0.37702 (6)	0.88600 (3)	0.04076 (14)	
N1	0.52635 (16)	0.32102 (16)	0.82195 (10)	0.0364 (4)	
N2	0.2542 (2)	0.4505 (2)	0.84783 (13)	0.0617 (6)	
N3	0.24283 (19)	0.47829 (19)	0.90328 (12)	0.0523 (5)	
N4	0.2266 (3)	0.5107 (3)	0.95606 (16)	0.0911 (9)	
N5	0.5950 (2)	0.0405 (2)	0.90254 (13)	0.0628 (6)	
N6	0.65128 (19)	0.0383 (2)	0.95230 (11)	0.0530 (5)	
N7	0.7116 (3)	0.0328 (3)	1.00051 (14)	0.0868 (9)	
N8	0.7028 (2)	0.4497 (2)	0.67838 (13)	0.0599 (6)	
N9	0.7403 (3)	0.5394 (3)	0.68504 (15)	0.0784 (7)	
N10	0.7844 (5)	0.6258 (4)	0.6907 (3)	0.164 (2)	
N11	0.17455 (15)	0.21022 (16)	0.67200 (9)	0.0348 (4)	
N12	0.14031 (15)	0.14605 (16)	0.62444 (9)	0.0364 (4)	
N13	0.34995 (16)	−0.00852 (16)	0.70729 (10)	0.0386 (4)	
N14	0.28322 (16)	−0.03796 (16)	0.65579 (10)	0.0376 (4)	
N15	0.41462 (16)	0.20726 (16)	0.58126 (9)	0.0366 (4)	
N16	0.34604 (16)	0.13737 (17)	0.54964 (9)	0.0391 (4)	
C1	0.5783 (3)	0.5462 (2)	0.86171 (17)	0.0630 (7)	
H1A	0.611474	0.578126	0.812136	0.094*	
H1B	0.623358	0.578596	0.893520	0.094*	
H1C	0.490828	0.569406	0.866928	0.094*	
C2	0.7669 (2)	0.3327 (3)	0.88212 (17)	0.0609 (7)	
H2A	0.776677	0.245323	0.897247	0.091*	
H2B	0.807741	0.368582	0.914024	0.091*	
H2C	0.804121	0.361215	0.833093	0.091*	
C3	0.5166 (3)	0.3129 (3)	0.97832 (14)	0.0679 (8)	
H3A	0.431839	0.345395	0.980566	0.102*	
H3B	0.561231	0.334619	1.014358	0.102*	
H3C	0.515542	0.225503	0.987630	0.102*	
C4	0.0628 (2)	0.3588 (2)	0.73746 (14)	0.0502 (6)	
H4A	−0.021138	0.392982	0.740781	0.075*	
H4B	0.120560	0.423202	0.717294	0.075*	
H4C	0.084442	0.315666	0.785218	0.075*	
C5	0.06979 (19)	0.2730 (2)	0.68965 (12)	0.0395 (5)	
C6	−0.0299 (2)	0.2471 (2)	0.65436 (13)	0.0473 (6)	
H6	−0.112202	0.277794	0.657710	0.057*	
C7	0.0164 (2)	0.1681 (2)	0.61406 (13)	0.0444 (5)	
C8	−0.0496 (2)	0.1139 (3)	0.56439 (17)	0.0670 (8)	
H8A	−0.137189	0.137596	0.567114	0.100*	
H8B	−0.040632	0.026449	0.578982	0.100*	
H8C	−0.013188	0.142877	0.515209	0.100*	
C9	0.4515 (3)	−0.1292 (2)	0.81859 (16)	0.0667 (8)	
H9A	0.529813	−0.088597	0.803667	0.100*	
H9B	0.467871	−0.214272	0.840157	0.100*	
H9C	0.403603	−0.093153	0.853637	0.100*	
C10	0.3789 (2)	−0.1161 (2)	0.75371 (13)	0.0441 (5)	
C11	0.3304 (2)	−0.2115 (2)	0.73203 (14)	0.0494 (6)	
H11	0.337355	−0.294313	0.754884	0.059*	
C12	0.2705 (2)	−0.1603 (2)	0.67069 (13)	0.0432 (5)	
C13	0.2017 (3)	−0.2218 (2)	0.62601 (17)	0.0620 (7)	
H13A	0.208509	−0.308701	0.644984	0.093*	
H13B	0.237628	−0.200302	0.576362	0.093*	
H13C	0.114672	−0.195770	0.627929	0.093*	
C14	0.5931 (2)	0.3525 (3)	0.53659 (14)	0.0548 (6)	
H14A	0.653782	0.306889	0.567743	0.082*	
H14B	0.553101	0.414548	0.558056	0.082*	
H14C	0.634626	0.390003	0.489801	0.082*	
C15	0.4963 (2)	0.2684 (2)	0.52753 (12)	0.0430 (5)	
C16	0.4801 (2)	0.2372 (3)	0.46324 (13)	0.0552 (6)	
H16	0.524464	0.266948	0.418171	0.066*	
C17	0.3864 (2)	0.1541 (3)	0.47857 (12)	0.0502 (6)	
C18	0.3358 (3)	0.0878 (3)	0.42916 (15)	0.0789 (10)	
H18A	0.339169	0.001538	0.451014	0.118*	
H18B	0.385699	0.104993	0.383157	0.118*	
H18C	0.250263	0.114463	0.421627	0.118*	
B1	0.2358 (2)	0.0624 (2)	0.59292 (13)	0.0387 (5)	
H1	0.194700	0.024994	0.560488	0.046*	
N17	−0.05817 (17)	0.82275 (17)	0.86189 (12)	0.0459 (5)	
C19	0.0230 (2)	0.7189 (2)	0.84364 (18)	0.0634 (7)	
H19A	0.058171	0.745072	0.793400	0.076*	
H19B	0.092600	0.703033	0.874210	0.076*	
C20	−0.0436 (3)	0.6007 (3)	0.8532 (2)	0.0762 (9)	
H20A	−0.113210	0.615106	0.823468	0.114*	
H20B	−0.073955	0.570685	0.903417	0.114*	
H20C	0.013988	0.541409	0.838652	0.114*	
C21	−0.1151 (3)	0.7881 (3)	0.94073 (15)	0.0603 (7)	
H21A	−0.166352	0.856816	0.950127	0.072*	
H21B	−0.170281	0.720491	0.946621	0.072*	
C22	−0.0203 (4)	0.7522 (4)	0.9976 (2)	0.1076 (13)	
H22A	0.042863	0.812729	0.987749	0.161*	
H22B	0.018634	0.674436	0.995761	0.161*	
H22C	−0.062494	0.746732	1.045169	0.161*	
C23	0.0264 (2)	0.9312 (3)	0.84888 (19)	0.0695 (8)	
H23A	0.093986	0.908884	0.880721	0.083*	
H23B	0.064055	0.948195	0.799003	0.083*	
C24	−0.0391 (3)	1.0463 (3)	0.8616 (2)	0.0892 (11)	
H24A	−0.109205	1.066948	0.832393	0.134*	
H24B	0.018762	1.111840	0.848318	0.134*	
H24C	−0.068571	1.033470	0.912190	0.134*	
C25	−0.1686 (2)	0.8524 (3)	0.81586 (16)	0.0618 (7)	
H25A	−0.219688	0.917170	0.830344	0.074*	
H25B	−0.219841	0.780891	0.826174	0.074*	
C26	−0.1337 (4)	0.8915 (3)	0.73461 (19)	0.1038 (13)	
H26A	−0.208837	0.907254	0.709485	0.156*	
H26B	−0.083986	0.827723	0.719386	0.156*	
H26C	−0.086142	0.964464	0.723342	0.156*	

### Atomic displacement parameters (Å^2^)

**Table:**

	*U*^11^	*U*^22^	*U*^33^	*U*^12^	*U*^13^	*U*^23^
Mo1	0.02915 (9)	0.03079 (9)	0.02823 (9)	0.00145 (6)	−0.00491 (6)	−0.01069 (7)
Fe1	0.03339 (15)	0.03888 (16)	0.03479 (16)	0.00628 (12)	−0.00428 (12)	−0.01577 (13)
Fe2	0.03843 (16)	0.03578 (16)	0.03790 (17)	0.00523 (12)	−0.01024 (13)	−0.01174 (13)
Fe3	0.03283 (15)	0.03801 (16)	0.03821 (17)	−0.00029 (12)	−0.00218 (12)	−0.01491 (13)
S1	0.0392 (3)	0.0329 (3)	0.0357 (3)	0.0027 (2)	−0.0061 (2)	−0.0085 (2)
S2	0.0382 (3)	0.0434 (3)	0.0311 (3)	−0.0030 (2)	−0.0026 (2)	−0.0077 (2)
S3	0.0333 (3)	0.0422 (3)	0.0451 (3)	0.0073 (2)	−0.0064 (2)	−0.0211 (2)
Si1	0.0429 (3)	0.0444 (3)	0.0423 (3)	0.0074 (3)	−0.0129 (3)	−0.0228 (3)
N1	0.0354 (9)	0.0407 (10)	0.0389 (10)	0.0042 (7)	−0.0080 (7)	−0.0199 (8)
N2	0.0562 (12)	0.0772 (15)	0.0643 (14)	0.0234 (11)	−0.0097 (11)	−0.0443 (13)
N3	0.0475 (11)	0.0576 (13)	0.0591 (13)	0.0150 (9)	−0.0091 (10)	−0.0297 (11)
N4	0.096 (2)	0.120 (2)	0.0768 (18)	0.0343 (18)	−0.0206 (15)	−0.0637 (18)
N5	0.0733 (15)	0.0528 (13)	0.0634 (15)	0.0100 (11)	−0.0338 (12)	−0.0089 (11)
N6	0.0509 (12)	0.0603 (13)	0.0405 (11)	0.0139 (10)	−0.0056 (9)	−0.0021 (10)
N7	0.0888 (19)	0.114 (2)	0.0518 (15)	0.0193 (17)	−0.0253 (14)	−0.0073 (15)
N8	0.0541 (12)	0.0585 (14)	0.0704 (15)	−0.0208 (11)	0.0061 (11)	−0.0223 (12)
N9	0.098 (2)	0.0624 (16)	0.0773 (18)	−0.0253 (15)	−0.0151 (15)	−0.0145 (14)
N10	0.239 (5)	0.104 (3)	0.165 (4)	−0.085 (3)	−0.032 (4)	−0.039 (3)
N11	0.0312 (8)	0.0413 (10)	0.0346 (9)	0.0004 (7)	−0.0053 (7)	−0.0139 (8)
N12	0.0334 (9)	0.0415 (10)	0.0381 (10)	−0.0005 (7)	−0.0106 (7)	−0.0144 (8)
N13	0.0447 (10)	0.0340 (9)	0.0401 (10)	0.0009 (8)	−0.0124 (8)	−0.0118 (8)
N14	0.0390 (9)	0.0377 (10)	0.0410 (10)	−0.0006 (7)	−0.0081 (8)	−0.0174 (8)
N15	0.0383 (9)	0.0442 (10)	0.0300 (9)	−0.0022 (8)	−0.0035 (7)	−0.0138 (8)
N16	0.0418 (10)	0.0484 (11)	0.0327 (9)	−0.0032 (8)	−0.0058 (7)	−0.0189 (8)
C1	0.0721 (18)	0.0512 (15)	0.0770 (19)	0.0073 (13)	−0.0146 (15)	−0.0358 (14)
C2	0.0464 (14)	0.0615 (17)	0.080 (2)	0.0057 (12)	−0.0200 (13)	−0.0239 (15)
C3	0.0783 (19)	0.086 (2)	0.0428 (15)	0.0183 (16)	−0.0071 (13)	−0.0261 (14)
C4	0.0371 (12)	0.0598 (15)	0.0596 (15)	0.0097 (11)	−0.0028 (11)	−0.0287 (13)
C5	0.0325 (10)	0.0442 (12)	0.0419 (12)	0.0022 (9)	−0.0034 (9)	−0.0117 (10)
C6	0.0305 (11)	0.0581 (15)	0.0547 (14)	0.0047 (10)	−0.0090 (10)	−0.0159 (12)
C7	0.0356 (11)	0.0502 (13)	0.0494 (13)	−0.0029 (10)	−0.0129 (10)	−0.0121 (11)
C8	0.0521 (15)	0.081 (2)	0.081 (2)	−0.0002 (14)	−0.0302 (14)	−0.0354 (17)
C9	0.099 (2)	0.0361 (13)	0.0663 (18)	0.0066 (13)	−0.0411 (16)	−0.0041 (12)
C10	0.0522 (13)	0.0347 (11)	0.0473 (13)	0.0011 (10)	−0.0111 (10)	−0.0114 (10)
C11	0.0583 (14)	0.0310 (11)	0.0597 (15)	0.0003 (10)	−0.0100 (12)	−0.0111 (11)
C12	0.0421 (12)	0.0377 (12)	0.0549 (14)	−0.0028 (9)	−0.0024 (10)	−0.0216 (11)
C13	0.0659 (17)	0.0518 (15)	0.081 (2)	−0.0043 (13)	−0.0172 (15)	−0.0343 (14)
C14	0.0540 (14)	0.0683 (17)	0.0406 (13)	−0.0176 (13)	0.0077 (11)	−0.0123 (12)
C15	0.0405 (12)	0.0547 (14)	0.0344 (12)	−0.0026 (10)	−0.0004 (9)	−0.0133 (10)
C16	0.0565 (15)	0.0805 (19)	0.0298 (12)	−0.0107 (13)	0.0057 (10)	−0.0177 (12)
C17	0.0519 (14)	0.0704 (17)	0.0334 (12)	−0.0046 (12)	−0.0034 (10)	−0.0220 (12)
C18	0.094 (2)	0.112 (3)	0.0445 (16)	−0.026 (2)	−0.0062 (15)	−0.0395 (17)
B1	0.0408 (13)	0.0443 (14)	0.0372 (13)	−0.0007 (10)	−0.0088 (10)	−0.0197 (11)
N17	0.0336 (9)	0.0420 (10)	0.0625 (13)	0.0003 (8)	−0.0035 (9)	−0.0145 (9)
C19	0.0444 (14)	0.0592 (17)	0.092 (2)	0.0125 (12)	−0.0051 (14)	−0.0315 (15)
C20	0.0679 (18)	0.0555 (17)	0.117 (3)	0.0133 (14)	−0.0203 (18)	−0.0416 (18)
C21	0.0664 (17)	0.0541 (16)	0.0604 (17)	−0.0067 (13)	0.0031 (13)	−0.0165 (13)
C22	0.145 (4)	0.109 (3)	0.078 (2)	0.006 (3)	−0.047 (2)	−0.028 (2)
C23	0.0474 (15)	0.0598 (17)	0.104 (2)	−0.0187 (13)	0.0132 (15)	−0.0286 (17)
C24	0.077 (2)	0.0534 (18)	0.138 (3)	−0.0241 (16)	0.021 (2)	−0.033 (2)
C25	0.0562 (15)	0.0550 (16)	0.0770 (19)	0.0117 (12)	−0.0220 (14)	−0.0183 (14)
C26	0.155 (4)	0.082 (3)	0.074 (2)	0.013 (2)	−0.034 (2)	−0.013 (2)

### Geometric parameters (Å, º)

**Table:**

Mo1—Fe1	2.7743 (12)	Fe1—N2	1.937 (2)
Mo1—Fe2	2.8012 (13)	Fe2—Fe3	2.6286 (11)
Mo1—Fe3	2.7920 (11)	Fe2—S2	2.2906 (14)
Mo1—S1	2.3660 (15)	Fe2—S3	2.2923 (13)
Mo1—S2	2.3638 (13)	Fe2—N1	1.917 (2)
Mo1—S3	2.3758 (14)	Fe2—N5	1.932 (2)
Fe1—Fe2	2.6368 (12)	Fe3—S1	2.2824 (12)
Fe1—Fe3	2.6123 (12)	Fe3—S3	2.2784 (14)
Fe1—S1	2.2794 (14)	Fe3—N1	1.936 (2)
Fe1—S2	2.2678 (14)	Fe3—N8	1.922 (2)
Fe1—N1	1.9386 (19)	Si1—N1	1.7530 (19)
			
Fe1—Mo1—Fe2	56.45 (3)	C17—N16—B1	128.75 (18)
Fe1—Mo1—Fe3	55.98 (2)	Si1—C1—H1A	109.5
Fe3—Mo1—Fe2	56.06 (2)	Si1—C1—H1B	109.5
S1—Mo1—Fe1	51.91 (3)	Si1—C1—H1C	109.5
S1—Mo1—Fe2	96.584 (17)	H1A—C1—H1B	109.5
S1—Mo1—Fe3	51.73 (3)	H1A—C1—H1C	109.5
S1—Mo1—S3	101.05 (2)	H1B—C1—H1C	109.5
S2—Mo1—Fe1	51.63 (3)	Si1—C2—H2A	109.5
S2—Mo1—Fe2	51.81 (4)	Si1—C2—H2B	109.5
S2—Mo1—Fe3	95.96 (3)	Si1—C2—H2C	109.5
S2—Mo1—S1	101.54 (2)	H2A—C2—H2B	109.5
S2—Mo1—S3	101.72 (3)	H2A—C2—H2C	109.5
S3—Mo1—Fe1	96.298 (18)	H2B—C2—H2C	109.5
S3—Mo1—Fe2	51.77 (2)	Si1—C3—H3A	109.5
S3—Mo1—Fe3	51.56 (3)	Si1—C3—H3B	109.5
N11—Mo1—Fe1	98.22 (4)	Si1—C3—H3C	109.5
N11—Mo1—Fe2	139.46 (4)	H3A—C3—H3B	109.5
N11—Mo1—Fe3	139.11 (5)	H3A—C3—H3C	109.5
N11—Mo1—S1	87.57 (5)	H3B—C3—H3C	109.5
N11—Mo1—S2	87.78 (5)	H4A—C4—H4B	109.5
N11—Mo1—S3	165.47 (5)	H4A—C4—H4C	109.5
N11—Mo1—N13	81.98 (6)	H4B—C4—H4C	109.5
N13—Mo1—Fe1	136.92 (5)	C5—C4—H4A	109.5
N13—Mo1—Fe2	96.16 (4)	C5—C4—H4B	109.5
N13—Mo1—Fe3	138.87 (5)	C5—C4—H4C	109.5
N13—Mo1—S1	167.20 (4)	N11—C5—C4	125.30 (19)
N13—Mo1—S2	85.49 (5)	N11—C5—C6	109.4 (2)
N13—Mo1—S3	87.79 (5)	C6—C5—C4	125.25 (19)
N15—Mo1—Fe1	140.00 (5)	C5—C6—H6	126.6
N15—Mo1—Fe2	138.97 (5)	C7—C6—C5	106.85 (19)
N15—Mo1—Fe3	99.00 (5)	C7—C6—H6	126.6
N15—Mo1—S1	88.24 (5)	N12—C7—C6	107.88 (19)
N15—Mo1—S2	165.03 (5)	N12—C7—C8	123.1 (2)
N15—Mo1—S3	87.27 (5)	C6—C7—C8	129.0 (2)
N15—Mo1—N11	81.28 (6)	C7—C8—H8A	109.5
N15—Mo1—N13	82.90 (6)	C7—C8—H8B	109.5
Fe2—Fe1—Mo1	62.29 (3)	C7—C8—H8C	109.5
Fe3—Fe1—Mo1	62.35 (2)	H8A—C8—H8B	109.5
Fe3—Fe1—Fe2	60.10 (3)	H8A—C8—H8C	109.5
S1—Fe1—Mo1	54.78 (4)	H8B—C8—H8C	109.5
S1—Fe1—Fe2	103.55 (2)	H9A—C9—H9B	109.5
S1—Fe1—Fe3	55.12 (2)	H9A—C9—H9C	109.5
S2—Fe1—Mo1	54.81 (3)	H9B—C9—H9C	109.5
S2—Fe1—Fe2	55.06 (4)	C10—C9—H9A	109.5
S2—Fe1—Fe3	103.60 (2)	C10—C9—H9B	109.5
S2—Fe1—S1	107.36 (2)	C10—C9—H9C	109.5
N1—Fe1—Mo1	95.61 (5)	N13—C10—C9	124.7 (2)
N1—Fe1—Fe2	46.51 (6)	N13—C10—C11	109.7 (2)
N1—Fe1—Fe3	47.58 (6)	C11—C10—C9	125.6 (2)
N1—Fe1—S1	101.71 (6)	C10—C11—H11	126.6
N1—Fe1—S2	100.35 (6)	C12—C11—C10	106.9 (2)
N2—Fe1—Mo1	151.18 (7)	C12—C11—H11	126.6
N2—Fe1—Fe2	140.51 (8)	N14—C12—C11	107.39 (19)
N2—Fe1—Fe3	138.19 (8)	N14—C12—C13	123.8 (2)
N2—Fe1—S1	114.78 (8)	C11—C12—C13	128.8 (2)
N2—Fe1—S2	117.42 (9)	C12—C13—H13A	109.5
N2—Fe1—N1	113.18 (9)	C12—C13—H13B	109.5
Fe1—Fe2—Mo1	61.262 (19)	C12—C13—H13C	109.5
Fe3—Fe2—Mo1	61.79 (3)	H13A—C13—H13B	109.5
Fe3—Fe2—Fe1	59.49 (3)	H13A—C13—H13C	109.5
S2—Fe2—Mo1	54.20 (2)	H13B—C13—H13C	109.5
S2—Fe2—Fe1	54.26 (3)	H14A—C14—H14B	109.5
S2—Fe2—Fe3	102.45 (2)	H14A—C14—H14C	109.5
S2—Fe2—S3	106.67 (3)	H14B—C14—H14C	109.5
S3—Fe2—Mo1	54.50 (4)	C15—C14—H14A	109.5
S3—Fe2—Fe1	102.32 (3)	C15—C14—H14B	109.5
S3—Fe2—Fe3	54.65 (3)	C15—C14—H14C	109.5
N1—Fe2—Mo1	95.26 (5)	N15—C15—C14	125.3 (2)
N1—Fe2—Fe1	47.19 (6)	N15—C15—C16	109.4 (2)
N1—Fe2—Fe3	47.30 (6)	C16—C15—C14	125.2 (2)
N1—Fe2—S2	100.22 (5)	C15—C16—H16	126.5
N1—Fe2—S3	100.87 (6)	C17—C16—C15	107.1 (2)
N1—Fe2—N5	114.41 (9)	C17—C16—H16	126.5
N5—Fe2—Mo1	150.13 (7)	N16—C17—C16	107.5 (2)
N5—Fe2—Fe1	143.49 (8)	N16—C17—C18	123.6 (2)
N5—Fe2—Fe3	137.79 (8)	C16—C17—C18	128.8 (2)
N5—Fe2—S2	119.35 (8)	C17—C18—H18A	109.5
N5—Fe2—S3	113.06 (8)	C17—C18—H18B	109.5
Fe1—Fe3—Mo1	61.67 (3)	C17—C18—H18C	109.5
Fe1—Fe3—Fe2	60.41 (2)	H18A—C18—H18B	109.5
Fe2—Fe3—Mo1	62.14 (3)	H18A—C18—H18C	109.5
S1—Fe3—Mo1	54.47 (4)	H18B—C18—H18C	109.5
S1—Fe3—Fe1	55.01 (4)	N12—B1—H1	109.3
S1—Fe3—Fe2	103.72 (3)	N14—B1—N12	108.87 (18)
S3—Fe3—Mo1	54.75 (3)	N14—B1—N16	110.20 (18)
S3—Fe3—Fe1	103.47 (2)	N14—B1—H1	109.3
S3—Fe3—Fe2	55.14 (4)	N16—B1—N12	109.76 (18)
S3—Fe3—S1	106.74 (2)	N16—B1—H1	109.3
N1—Fe3—Mo1	95.10 (6)	C19—N17—C21	111.1 (2)
N1—Fe3—Fe1	47.65 (5)	C19—N17—C23	106.64 (19)
N1—Fe3—Fe2	46.68 (6)	C23—N17—C21	110.8 (2)
N1—Fe3—S1	101.67 (6)	C25—N17—C19	111.1 (2)
N1—Fe3—S3	100.75 (6)	C25—N17—C21	105.62 (19)
N8—Fe3—Mo1	151.35 (8)	C25—N17—C23	111.6 (2)
N8—Fe3—Fe1	139.18 (7)	N17—C19—H19A	108.5
N8—Fe3—Fe2	139.85 (8)	N17—C19—H19B	108.5
N8—Fe3—S1	115.52 (8)	H19A—C19—H19B	107.5
N8—Fe3—S3	116.62 (8)	C20—C19—N17	115.3 (2)
N8—Fe3—N1	113.54 (9)	C20—C19—H19A	108.5
Fe1—S1—Mo1	73.32 (2)	C20—C19—H19B	108.5
Fe1—S1—Fe3	69.87 (3)	C19—C20—H20A	109.5
Fe3—S1—Mo1	73.81 (2)	C19—C20—H20B	109.5
Fe1—S2—Mo1	73.56 (2)	C19—C20—H20C	109.5
Fe1—S2—Fe2	70.68 (2)	H20A—C20—H20B	109.5
Fe2—S2—Mo1	73.98 (3)	H20A—C20—H20C	109.5
Fe2—S3—Mo1	73.73 (3)	H20B—C20—H20C	109.5
Fe3—S3—Mo1	73.690 (18)	N17—C21—H21A	108.5
Fe3—S3—Fe2	70.21 (2)	N17—C21—H21B	108.5
N1—Si1—C1	108.62 (11)	H21A—C21—H21B	107.5
N1—Si1—C2	108.68 (11)	C22—C21—N17	115.0 (3)
N1—Si1—C3	109.06 (13)	C22—C21—H21A	108.5
C1—Si1—C2	109.26 (13)	C22—C21—H21B	108.5
C1—Si1—C3	109.89 (14)	C21—C22—H22A	109.5
C2—Si1—C3	111.28 (14)	C21—C22—H22B	109.5
Fe1—N1—Fe2	86.29 (7)	C21—C22—H22C	109.5
Fe1—N1—Fe3	84.78 (7)	H22A—C22—H22B	109.5
Fe2—N1—Fe3	86.02 (7)	H22A—C22—H22C	109.5
Si1—N1—Fe1	128.04 (9)	H22B—C22—H22C	109.5
Si1—N1—Fe2	125.15 (10)	N17—C23—H23A	108.6
Si1—N1—Fe3	131.52 (11)	N17—C23—H23B	108.6
N3—N2—Fe1	141.17 (19)	H23A—C23—H23B	107.6
N4—N3—N2	176.0 (3)	C24—C23—N17	114.7 (2)
N6—N5—Fe2	142.0 (2)	C24—C23—H23A	108.6
N7—N6—N5	176.8 (3)	C24—C23—H23B	108.6
N9—N8—Fe3	138.5 (2)	C23—C24—H24A	109.5
N10—N9—N8	175.9 (5)	C23—C24—H24B	109.5
N12—N11—Mo1	119.02 (12)	C23—C24—H24C	109.5
C5—N11—Mo1	134.92 (14)	H24A—C24—H24B	109.5
C5—N11—N12	106.07 (16)	H24A—C24—H24C	109.5
N11—N12—B1	121.02 (16)	H24B—C24—H24C	109.5
C7—N12—N11	109.76 (17)	N17—C25—H25A	108.6
C7—N12—B1	129.20 (18)	N17—C25—H25B	108.6
N14—N13—Mo1	119.09 (12)	N17—C25—C26	114.8 (3)
C10—N13—Mo1	135.19 (14)	H25A—C25—H25B	107.5
C10—N13—N14	105.60 (17)	C26—C25—H25A	108.6
N13—N14—B1	120.39 (17)	C26—C25—H25B	108.6
C12—N14—N13	110.38 (17)	C25—C26—H26A	109.5
C12—N14—B1	129.22 (18)	C25—C26—H26B	109.5
N16—N15—Mo1	118.99 (13)	C25—C26—H26C	109.5
C15—N15—Mo1	134.89 (14)	H26A—C26—H26B	109.5
C15—N15—N16	106.07 (17)	H26A—C26—H26C	109.5
N15—N16—B1	121.19 (17)	H26B—C26—H26C	109.5
C17—N16—N15	109.92 (18)		
			
Mo1—N11—N12—C7	179.77 (14)	C3—Si1—N1—Fe3	−176.87 (13)
Mo1—N11—N12—B1	1.1 (2)	C4—C5—C6—C7	177.8 (2)
Mo1—N11—C5—C4	1.8 (4)	C5—N11—N12—C7	−0.8 (2)
Mo1—N11—C5—C6	−179.82 (16)	C5—N11—N12—B1	−179.43 (19)
Mo1—N13—N14—C12	−176.06 (14)	C5—C6—C7—N12	0.1 (3)
Mo1—N13—N14—B1	4.2 (2)	C5—C6—C7—C8	−178.2 (3)
Mo1—N13—C10—C9	−4.3 (4)	C7—N12—B1—N14	−117.7 (2)
Mo1—N13—C10—C11	175.44 (16)	C7—N12—B1—N16	121.7 (2)
Mo1—N15—N16—C17	−177.10 (15)	C9—C10—C11—C12	179.8 (3)
Mo1—N15—N16—B1	6.9 (2)	C10—N13—N14—C12	0.6 (2)
Mo1—N15—C15—C14	−0.3 (4)	C10—N13—N14—B1	−179.13 (19)
Mo1—N15—C15—C16	177.25 (17)	C10—C11—C12—N14	0.3 (3)
N11—N12—C7—C6	0.4 (3)	C10—C11—C12—C13	−179.4 (2)
N11—N12—C7—C8	178.9 (2)	C12—N14—B1—N12	116.7 (2)
N11—N12—B1—N14	60.7 (3)	C12—N14—B1—N16	−122.8 (2)
N11—N12—B1—N16	−60.0 (2)	C14—C15—C16—C17	177.1 (2)
N11—C5—C6—C7	−0.7 (3)	C15—N15—N16—C17	0.9 (2)
N12—N11—C5—C4	−177.5 (2)	C15—N15—N16—B1	−175.06 (19)
N12—N11—C5—C6	0.9 (2)	C15—C16—C17—N16	0.9 (3)
N13—N14—C12—C11	−0.6 (3)	C15—C16—C17—C18	−177.4 (3)
N13—N14—C12—C13	179.1 (2)	C17—N16—B1—N12	−119.9 (2)
N13—N14—B1—N12	−63.6 (2)	C17—N16—B1—N14	120.2 (2)
N13—N14—B1—N16	56.8 (2)	B1—N12—C7—C6	178.9 (2)
N13—C10—C11—C12	0.1 (3)	B1—N12—C7—C8	−2.6 (4)
N14—N13—C10—C9	179.9 (2)	B1—N14—C12—C11	179.1 (2)
N14—N13—C10—C11	−0.4 (3)	B1—N14—C12—C13	−1.2 (4)
N15—N16—C17—C16	−1.2 (3)	B1—N16—C17—C16	174.4 (2)
N15—N16—C17—C18	177.3 (3)	B1—N16—C17—C18	−7.1 (4)
N15—N16—B1—N12	55.3 (2)	C19—N17—C21—C22	58.4 (3)
N15—N16—B1—N14	−64.6 (2)	C19—N17—C23—C24	177.9 (3)
N15—C15—C16—C17	−0.4 (3)	C19—N17—C25—C26	−60.3 (3)
N16—N15—C15—C14	−177.8 (2)	C21—N17—C19—C20	59.0 (3)
N16—N15—C15—C16	−0.3 (3)	C21—N17—C23—C24	−61.0 (3)
C1—Si1—N1—Fe1	−59.18 (17)	C21—N17—C25—C26	179.1 (2)
C1—Si1—N1—Fe2	−175.96 (13)	C23—N17—C19—C20	179.9 (3)
C1—Si1—N1—Fe3	63.38 (16)	C23—N17—C21—C22	−60.0 (3)
C2—Si1—N1—Fe1	−177.95 (14)	C23—N17—C25—C26	58.6 (3)
C2—Si1—N1—Fe2	65.27 (16)	C25—N17—C19—C20	−58.3 (3)
C2—Si1—N1—Fe3	−55.39 (17)	C25—N17—C21—C22	179.0 (3)
C3—Si1—N1—Fe1	60.57 (17)	C25—N17—C23—C24	56.3 (4)
C3—Si1—N1—Fe2	−56.21 (15)		
