# Supplementary material for: Synthesis and crystal structure of the cluster (Et4N)[(Tp*)MoFe3S3(μ3-NSiMe3)(N3)3]
Source: Acta Crystallogr E Crystallogr Commun. 2024 May 31;80(Pt 6):691–4. doi: 10.1107/S2056989024004833 (PMC11151308; doi:10.1107/S2056989024004833)
Supplement: Supplementary file 3 [file e-80-00691-sup3.docx]

**Supporting information**

**Synthesis and crystal structure of the cluster (Et_4_N)[(Tp*)MoFe_3_S_3_(μ_3_-NSiMe_3_)(N_3_)_3_]**

Yue Li^§^, Jia Wei^§^, Jie Han and Xu-Dong Chen*

**
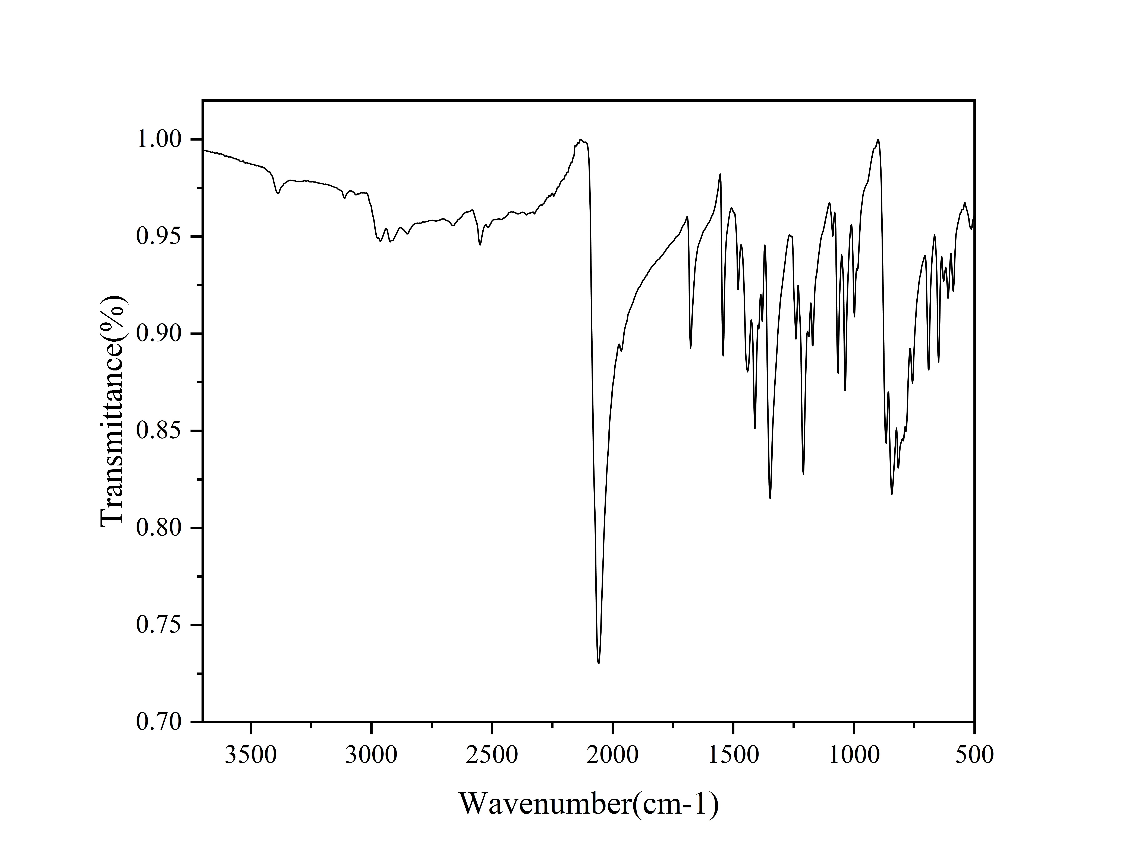
**

Figure S1. The IR spectrum of (Et_4_N)[(Tp*)MoFe_3_S_3_(μ_3_-NSiMe_3_)(N_3_)_3_].

**
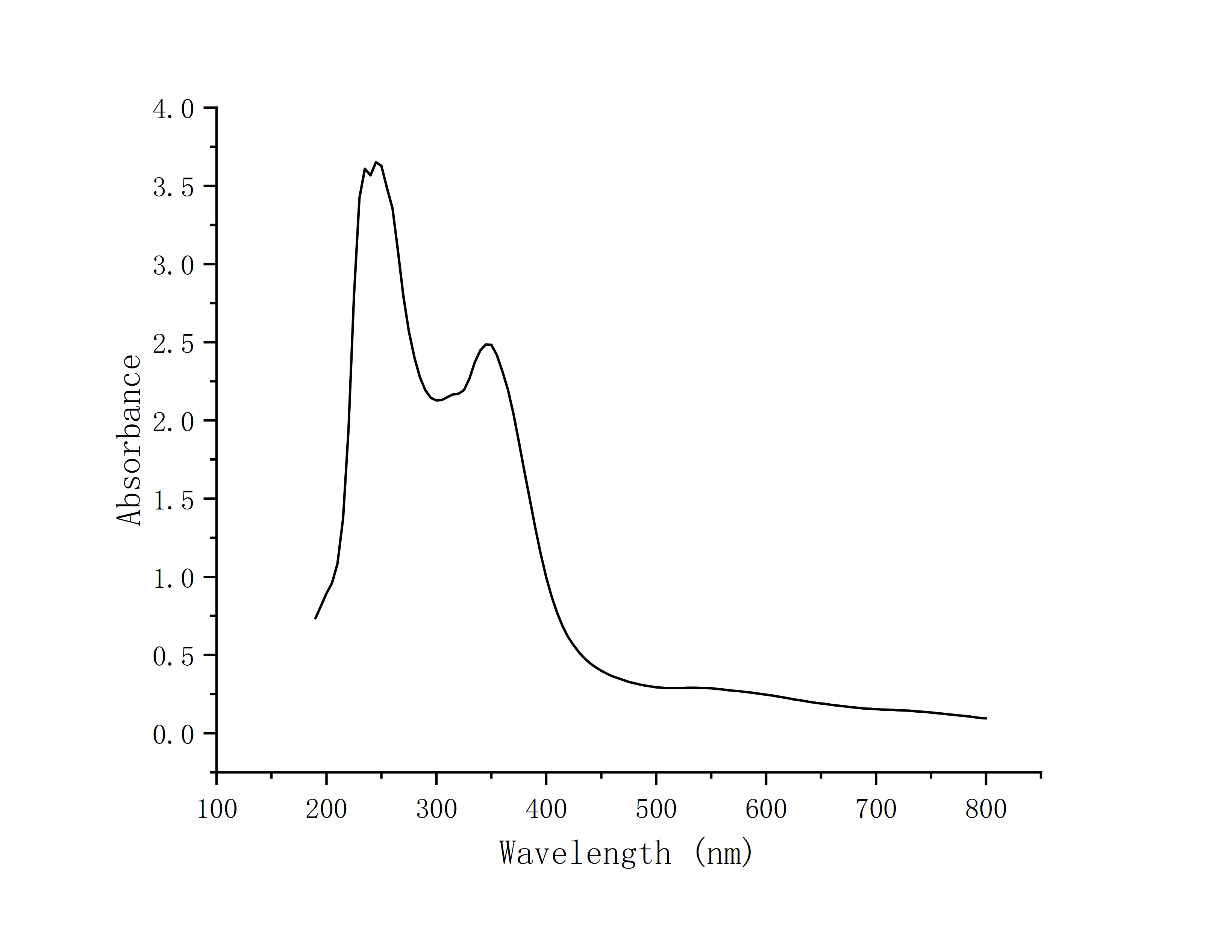
**

Figure S2. The UV-visible absorption spectroscopy of (Et_4_N)[(Tp*)MoFe_3_S_3_(μ_3_-NSiMe_3_)(N_3_)_3_].
